# Interferon Regulatory Factor 4 controls T_H1_ cell effector function and metabolism

**DOI:** 10.1038/srep35521

**Published:** 2016-10-20

**Authors:** Justus Mahnke, Valéa Schumacher, Stefanie Ahrens, Nadja Käding, Lea Marie Feldhoff, Magdalena Huber, Jan Rupp, Friederike Raczkowski, Hans-Willi Mittrücker

**Affiliations:** 1 Institute of Immunology, University Medical Center Hamburg-Eppendorf, Hamburg, Germany; 2 Department of Infectious Diseases and Microbiology, University of Lübeck, Lübeck, Germany; 3 Institute for Medical Microbiology and Hospital Hygiene, University of Marburg, Marburg, Germany

## Abstract

The transcription factor Interferon Regulatory Factor 4 (IRF4) is essential for T_H2_ and T_H17_ cell formation and controls peripheral CD8^+^ T cell differentiation. We used *Listeria monocytogenes* infection to characterize the function of IRF4 in T_H1_ responses. IRF4^−/−^ mice generated only marginal numbers of listeria-specific T_H1_ cells. After transfer into infected mice, IRF4^−/−^ CD4^+^ T cells failed to differentiate into T_H1_ cells as indicated by reduced T-bet and IFN-γ expression, and showed limited proliferation. Activated IRF4^−/−^ CD4^+^ T cells exhibited diminished uptake of the glucose analog 2-NBDG, limited oxidative phosphorylation and strongly reduced aerobic glycolysis. Insufficient metabolic adaptation contributed to the limited proliferation and T_H1_ differentiation of IRF4^−/−^ CD4^+^ T cells. Our study identifies IRF4 as central regulator of T_H1_ responses and cellular metabolism. We propose that this function of IRF4 is fundamental for the initiation and maintenance of all T_H_ cell responses.

The transcription factor Interferon Regulatory Factor 4 (IRF4) is expressed in various hematopoietic cells, including B and T cells but also different macrophage and dendritic cell subsets^1^,^2^,^3^,^4^,^5^,^6^,^7^. In B cells, IRF4 controls the germinal center reaction and high IRF4 expression is a prerequisite for plasma cell formation. As a consequence, antibodies are almost completely absent in IRF4-deficient mice^8^,^9^. Naive peripheral T cells express only low levels of IRF4. Upon T cell receptor stimulation, IRF4 is rapidly expressed and subsequently controls differentiation processes of these cells^1^,^8^,^10^,^11^. Deficiency of IRF4 in CD4^+^ T cells results in a complete block in the formation of T_H2_, T_H9_, T_H17_ and follicular T_H_ (T_FH_) cells^12^,^13^,^14^,^15^,^16^,^17^,^18^,^19^,^20^. Although IRF4-deficiency allows the generation of Foxp3^+^ T_reg_ cells, these cells are impaired in their suppressive functions^21^,^22^. IRF4 also controls peripheral CD8^+^ T cells differentiation. We and others could demonstrate that following antigen recognition, IRF4-deficient CD8^+^ T cells start to proliferate and to express effector molecules such as IFN-γ and granzyme B. However, IRF4-deficent cells cannot sustain proliferation and fail to upregulate effector molecules to the level observed in wild type CD8^+^ effector T cells. In line with these results, IRF4-deficient CD8^+^ T cells express reduced levels of transcription factors associated with CD8^+^ effector T cell formation including T-bet, BLIMP1 and ID2^8^,^11^,^23^,^24^,^25^,^26^,^27^.

In contrast to other IRF family members, IRF4 binds interferon stimulated response elements (ISRE) with low affinity. However, in cooperation with transcription factors of the Ets or AP-1 families, IRF4 is able to strongly bind to Ets-IRF composite elements (EICE) or AP-1-IRF composite elements (AICE), respectively^9^,^28^. Cooperative binding with the Ets proteins PU.1 and SpiB to EICE has been demonstrated for B cells and myeloid cells. However, both transcription factors are usually not expressed in T cells, indicating that interaction of IRF4 with EICE does not commonly occur in T cells^29^,^30^. In contrast, T cells express the AP-1 proteins BATF, JunB, JunD and c-Jun, and cooperative binding of IRF4 with heterodimers of BATF and Jun family members was demonstrated for T_H17_ cells and CD8^+^ T cells^29^,^30^,^31^.

Using mRNA expression studies and chromatin immune precipitation (ChIP), target genes for IRF4 have been determined for T_H17_ and CD8^+^ T cells. These targets include a large number of genes involved in T cell activation and differentiation^25^,^30^,^31^,^32^. Interestingly, IRF4 and BATF frequently bind to regulatory DNA regions outside the promotors. Therefore, it was proposed that IRF4 and BATF might act as pioneering factors that promote and sustain chromatin remodeling and enhance accessibility of genes for other transcription factors, including lineage-specific factors such as T-bet or RORγt^25^,^29^,^31^,^32^. In CD8^+^ T cells, IRF4 controls expression of transcription factors involved in effector cell differentiation including *Tbx21* (encoding T-bet), *Prdm1* (encoding BLIMP1), *Runx3* and *Tcf7* (encoding TCF-1), as well as effector proteins such as cytokines and cytolytic proteins^11^,^25^,^26^. IRF4 is also involved in the metabolic changes of CD8^+^ T cells following activation. Naive T cells show basal levels of glucose and amino acid uptake and mainly use oxidative phosphorylation and fatty acid oxidation for energy production. T cell activation causes enhanced nutrient uptake as well as increased aerobic glycolysis and glutaminolysis. These changes in the metabolic profile are necessary to provide energy and substrates for *de novo* synthesis of proteins, nucleic acids and lipids required for proliferation and effector protein production^33^,^34^,^35^,^36^. Metabolic changes are controlled by different transcription factors including HIF1α, FOXO1 and FOXO3. IRF4 modulates the expression of these factors but also directly enhances expression of several proteins involved in nutrient uptake and glycolysis^25^,^33^. Impaired adaptation to metabolic demands can explain the failure of IRF4-deficient CD8^+^ T cells to sustain proliferation and to develop into mature effector cells^25^,^33^. IRF4 expression levels correlate with the strength of the TCR signal, thereby IRF4 links TCR affinity with the extent of metabolic changes following CD8^+^ T cell activation. It has been proposed that during immune responses this mechanism promotes the preferential expansion of high affinity CD8^+^ T cell populations^25^,^27^,^33^.

In contrast to its function in CD8^+^ T cells and CD4^+^ T_H_ cell subsets described above, the role IRF4 in T_H1_ cell development is less clear. T_H1_ differentiation of IRF4^−/−^ CD4^+^ T cells has been analyzed *in vitro* and *in vivo* using the *Leishmania major* infection model, with inconsistent results^12^,^13^,^14^,^17^. Although all studies provided evidence for T_H1_ differentiation of IRF4^−/−^ CD4^+^ T cells, the efficacy of this process ranged from marginal to close to that observed in WT cells.

Here, we use the *Listeria monocytogenes* infection model to analyze the role of IRF4 in T_H1_ cell differentiation and function. We demonstrate that IRF4 is crucial for the generation of a T_H1_ response. IRF4^−/−^ CD4^+^ T cells showed impaired T_H1_ differentiation and only limited proliferation after *in vitr*o and *in vivo* activation. Compared to control cells, activated IRF4^−/−^ CD4^+^ T cells exhibited impaired aerobic glycolysis and oxidative phosphorylation and this restricted metabolism could be responsible for the poor response of these cells.

## Results

### IRF4-deficient mice fail to mount a T_H1_ response to *Listeria monocytogenes*

**Figure 1:**
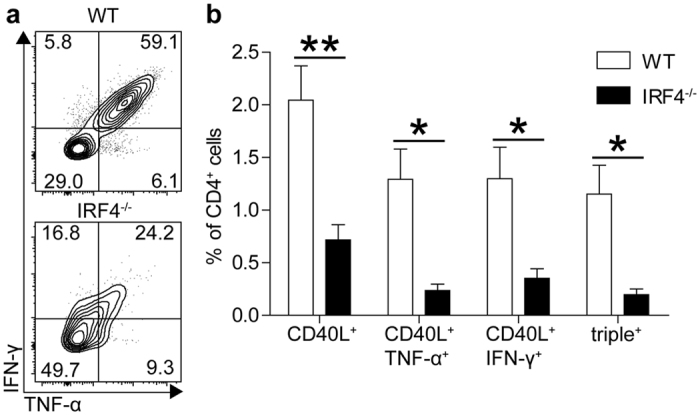
IRF4^−/−^ mice fail to mount a T_H1_ response against *L. monocytogenes.* WT and IRF4^−/−^ mice were i.v. infected with 5 × 10^4^ LmOVA. Eight days post infection, spleen cells were stimulated for 4 h with 10^−5^ M LLO_189-201_ and intracellular expression of IFN-γ, TNF-α and CD40L was determined by flow cytometry. (**a**) Representative dot plots for CD4^+^CD40L^+^ spleen cells from infected mice stimulated with LLO_189-201_. (**b**) Frequencies of CD40L^+^, IFN-γ^+^ and TNF-α^+^ cells among CD4^+^ T cells from spleens of infected mice following stimulation. Bars represent mean ± SEM from 4 individually analyzed mice per group. The result is representative for 4 independent experiments.

Wild type (WT) and IRF4^−/−^ mice were infected with LmOVA, a *L. monocytogenes* strain recombinant for ovalbumin^37^, and after 8 days the CD4^+^ T_H1_ response was analyzed. In WT mice, about half of the CD4^+^ T cells presented an activated CD44^+^CD62L^−^ phenotype (Figure S1). IRF4^−/−^ mice also accumulated CD62L^−^ CD4^+^ T cells, however, these cells failed to up-regulate CD44. CD4^+^ T cells from infected mice were stimulated with the immunodominant listeriolysin O peptide LLO_189-201_ and the expression of CD40L (CD154)^38^, IFN-γ and TNF-α was determined by intracellular staining (Fig. 1 and Figures S2 and S3). Following LLO_189-201_ stimulation, approx. 2% of CD4^+^ T cells from infected WT mice responded with up-regulation of CD40L and more than half of these cells co-expressed high levels of IFN-γ and TNF-α, a hallmark of T_H1_ cells. LLO_189-201_–specific CD4^+^ T cells were significantly reduced in IRF4^−/−^ mice, as indicated by the reduced frequencies of CD40L^+^ cells. Only a small fraction of these CD40L^+^ cells co-expressed IFN-γ and TNF-α and particularly IFN-γ^+^TNF-α^+^ cells were substantially reduced in IRF4^−/−^ mice. In addition, cytokine-positive IRF4^−/−^ CD4^+^ T cells displayed diminished staining intensity for IFN-γ and TNF-α, indicating that cells produced only low amounts of the cytokines (Fig. 1a,b).

### IRF4^−/−^ CD4^+^ T cells have an intrinsicT_H1_ differentiation defect

**Figure 2:**
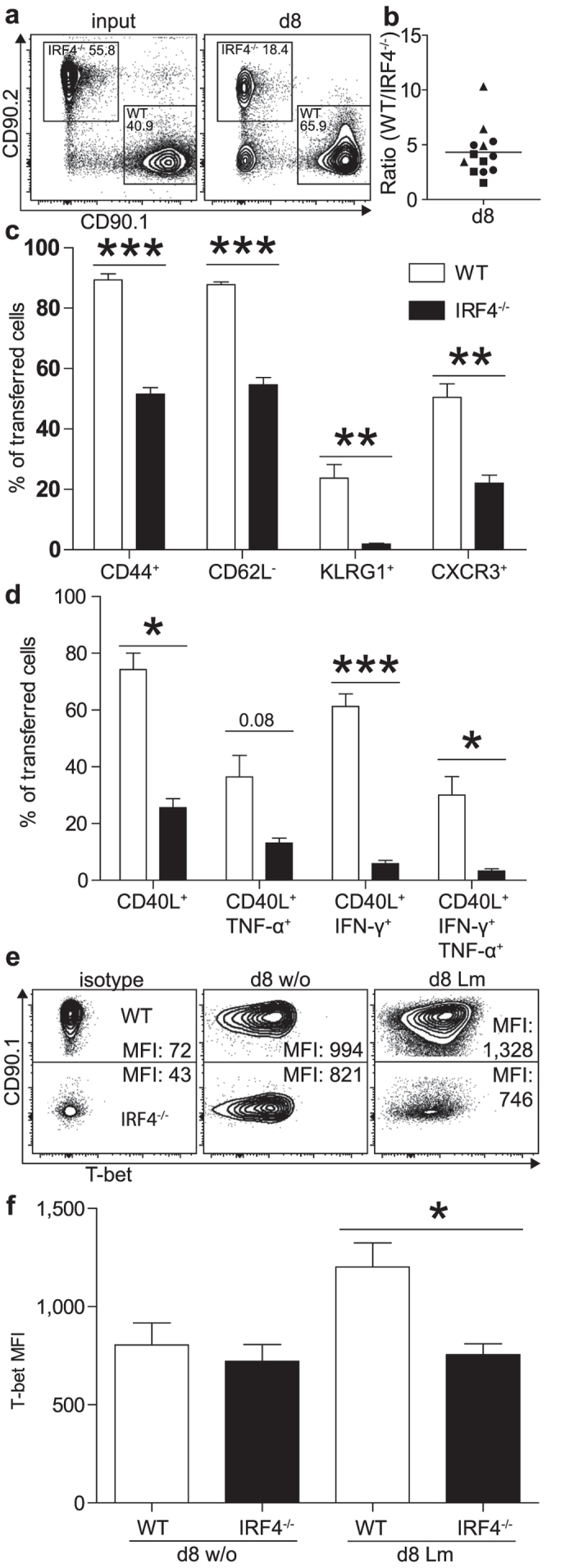
Impaired T_H1_ differentiation of IRF4^−/−^ CD4^+^ T cells. Purified CD4^+^ T cells from CD90.1^+^CD90.2^−^ WT and CD90.1^−^CD90.2^+^ IRF4^−/−^ mice were mixed in a 1:1 ratio and 2 × 10^6^ cells were i.v. transferred into RAG1^−/−^ mice, which had been infected with 10^5^ Lm 4 h earlier. Eight days post infection, spleen cells of mice were analyzed. (**a**) Representative CD90.1/CD90.2 dot blots of CD4^+^ T cells at the time point of transfer (input) and at d8 post infection. (**b**) Normalized ratio of WT and IRF4^−/−^ CD4^+^ T cells. The figure shows results for individual mice combined from 3 experiments and the mean. (**c**) Surface expression of CD44, CD62L, KLRG1 and CXCR3 on transferred CD4^+^ T cells. (**d**) CD40L, TNF-α, and IFN-γ expression of transferred CD4^+^ T cells from spleens of infected mice following stimulation with PMA/ionomycin. (**e**) Representative intracellular T-bet staining for transferred cells (MFI mean fluorescence intensity). (**f**) T-bet expression in WT and IRF4^−/−^ CD4^+^ T cells following transfer and with or without infection. (**c,d,f**) Show mean ± SEM for 4 individually analyzed mice per group. Results are representative for 3 independent experiments (with the exception of results for CXCR3 which are derived from one experiment only).

IRF4 is also expressed in subsets of dendritic cells and macrophages^2^,^3^,^4^,^5^,^6^,^7^ and absence of IRF4 in these cells could also impair CD4^+^ T cell responses. Therefore, CD4^+^ T cells were purified from WT and IRF4^−/−^ mice on a CD90.1^+^CD90.2^−^ and CD90.1^−^CD90.2^+^ background, respectively. Cells were mixed at a 1:1 ratio and co-transferred into RAG1^−/−^ mice, which had been infected with Lm 4h earlier. After 8 days, WT CD4^+^ T cells had outnumbered the IRF4^−/−^ CD4^+^ T cells by a factor of 4 (Fig. 2a,b). WT CD4^+^ T cells had uniformly acquired a CD44^+^CD62L^−^CD127^−^ phenotype (Fig. 2c and Figure S4a) and significant subpopulations expressed KLRG1 and the chemokine receptor CXCR3, which have been associated with T_H1_ cells. In contrast, IRF4^−/−^ CD4^+^ T cells showed reduced up-regulation of CD44 and CXCR3 and reduced down-regulation of CD62L and CD127. We failed to detect KLRG1^+^ IRF4^−/−^ CD4^+^ T cells. Following polyclonal stimulation, approx. 70% of transferred WT CD4^+^ T cells responded with CD40L expression and about 30% of CD4^+^ T cells had differentiated into IFN-γ^+^TNF-α^+^ T_H1_ cells (Fig. 2d and Figure S4b). In contrast, the IRF4^−/−^ CD4^+^ T cells showed reduced up-regulation of CD40L and only a small population was IFN-γ^+^TNF-α^+^. Finally, expression of the transcription factor T-bet was determined by intracellular staining (Fig. 2e,f). Whereas WT CD4^+^ T cells displayed elevated T-bet expression following infection, IRF4^−/−^ CD4^+^ T cells presented with expression levels comparable to those of cells from uninfected mice.

**Figure 3:**
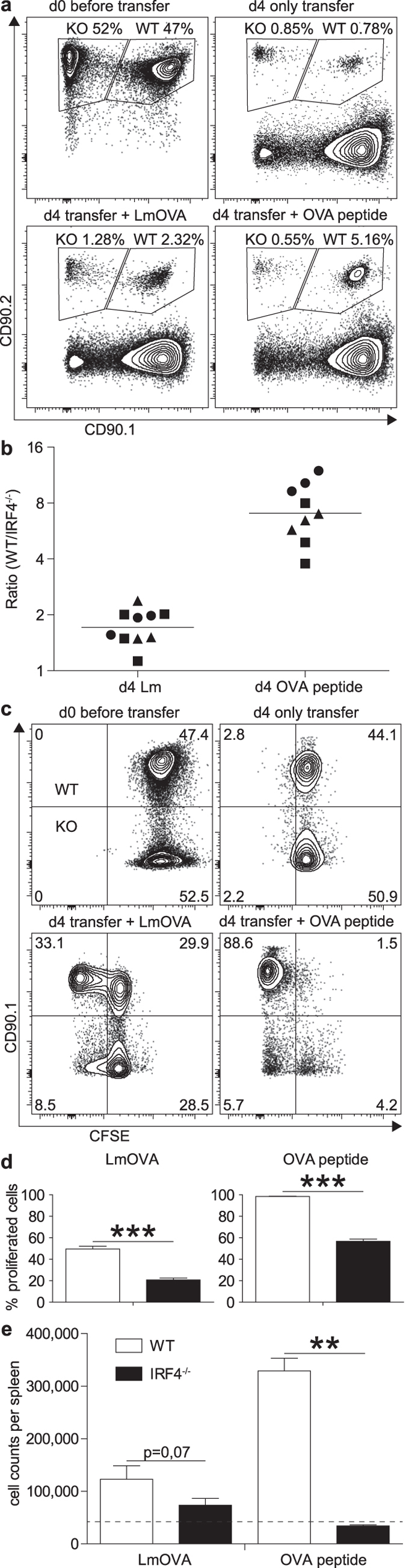
Impaired T cell response of IRF4^−/−^ CD4^+^ T cells following *in vivo* activation. Purified CD4^+^ T cells from WT CD90.1^+^CD90.2^+^ OT-II mice and IRF4^−/−^ CD90.1^−^CD90.2^+^ OT-II mice were mixed in a 1:1 ratio and labelled with CFSE. 3 × 10^6^ cells were i.v. transferred into CD90.1^+^CD90.2^−^ WT mice. Recipient mice had either been i.v. infected with 10^5^ LmOVA one day before or were i.v. treated with 50 μg OVA_323-339_ peptide and 30 μg LPS 1 h post transfer. Mice were analyzed 4d post transfer. (**a**) Representative CD90.1/CD90.2 dot plots of CD4-gated cells. (**b**) Normalized ratio of WT and IRF4^−/−^ CD4^+^ T cells. The figure shows results for individual mice combined from 3 experiments and the mean. (**c**) Representative CFSE/CD90.1 dot plots of CD4^+^CD90.2^+^ cells. (**d**) %-values of CFSE^low^ cells among transferred OT-II cells. (**e**) Total numbers of transferred WT and IRF4^−/−^ OT-II cells recovered from recipient mice. (dotted line: mean numbers of transferred cells recovered from mice without infection.) (**d**,**e**) Show mean ± SEM for 3 individually analyzed mice per group. Results are representative for 3 independent experiments.

**Figure 4:**
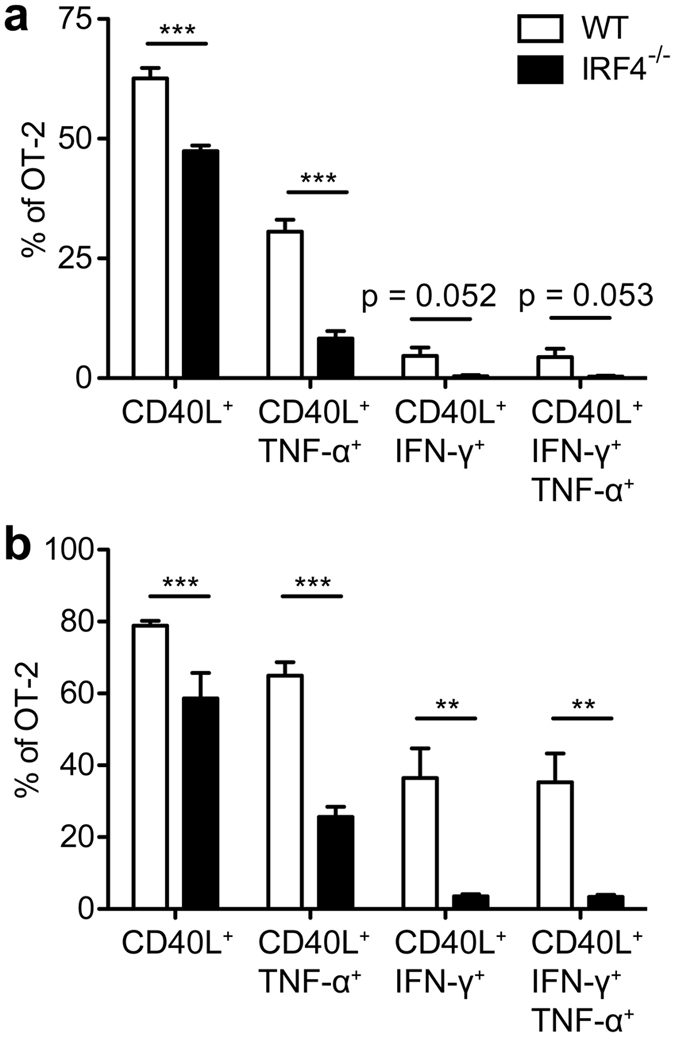
Reduced T_H1_ differentiation of transferred IRF4^−/−^ OT-II T cells. Purified CD4^+^ T cells from WT CD90.1^+^CD90.2^+^ or IRF4^−/−^ CD90.1^−^CD90.2^+^ OT-II mice were mixed in a 1:1 ratio. 10^6^ cells were i.v. transferred into CD90.1^+^CD90.2^−^ WT mice which had been i.v. infected with 10^5^ LmOVA one day before. On d4 post transfer, spleen cells from infected recipients were incubated for 4 h with 10^−6^M of OVA_323-339_ peptide or with PMA/ionomycin and stained intracellularly for CD40L, TNF-α, and IFN-γ. Figures show frequencies of CD40L^+^, IFN-γ^+^ and TNF-α^+^ cells among transferred OT-II T cells following stimulation with OVA_323-339_ (**a**) and PMA/ionomycin (**b**). Bars represent mean ± SEM from 6 individually analyzed mice per group. The result is representative for 4 independent experiments.

To characterize the specific CD4^+^ T cell response in immune competent mice, ovalbumin-specific CD4^+^ T cells from WT and IRF4^−/−^ OT-II mice (CD90.1^+^CD90.2^+^ and CD90.1^−^CD90.2^+^, respectively) were CFSE-labelled and competitively transferred into WT mice (CD90.1^+^CD90.2^−^). Recipients had either been infected with LmOVA or were treated with OVA_323-339_ peptide and LPS. Four days post transfer, WT OT-II cells had 2fold or 8fold outnumbered IRF4^−/−^ OT-II cells in mice infected with LmOVA or treated with OVA_323-339_ peptide, respectively (Fig. 3a,b). Accordingly, transferred IRF4^−/−^ OT-II cells remained to a larger extend CFSE-positive and this cell population failed to expand in infected or peptide-treated animals (Fig. 3c–e). In recipients infected with LmOVA, transferred WT OT-II cells expressed enhanced levels of IRF4 (Figure S5a). Compared to transferred WT OT-II cells, transferred IRF4^−/−^ OT-II cells showed diminished CD44 and KLRG1 up-regulation and CD62L down-regulation (Figure S5b). Spleen cells from LmOVA infected recipients were stimulated either with OVA_323-339_ peptide or with PMA/ionomycin and analyzed for the expression of CD40L and cytokines (Fig. 4a,b). With both stimuli, we observed a population of CD40L^+^IFN-γ^+^TNF-α^+^ T_H1_ cells among transferred WT OT-II cells, which was hardly detectable in IRF4^−/−^ OT-II cells. In conclusion, T cell transfer assays indicate that IRF4^−/−^ CD4^+^ T cells failed to respond to different stimuli *in vivo* with the generation of T_H1_ cells.

### IRF4^−/−^ CD4^+^ T cells show reduced T_H1_ differentiation *in vitro*

**Figure 5:**
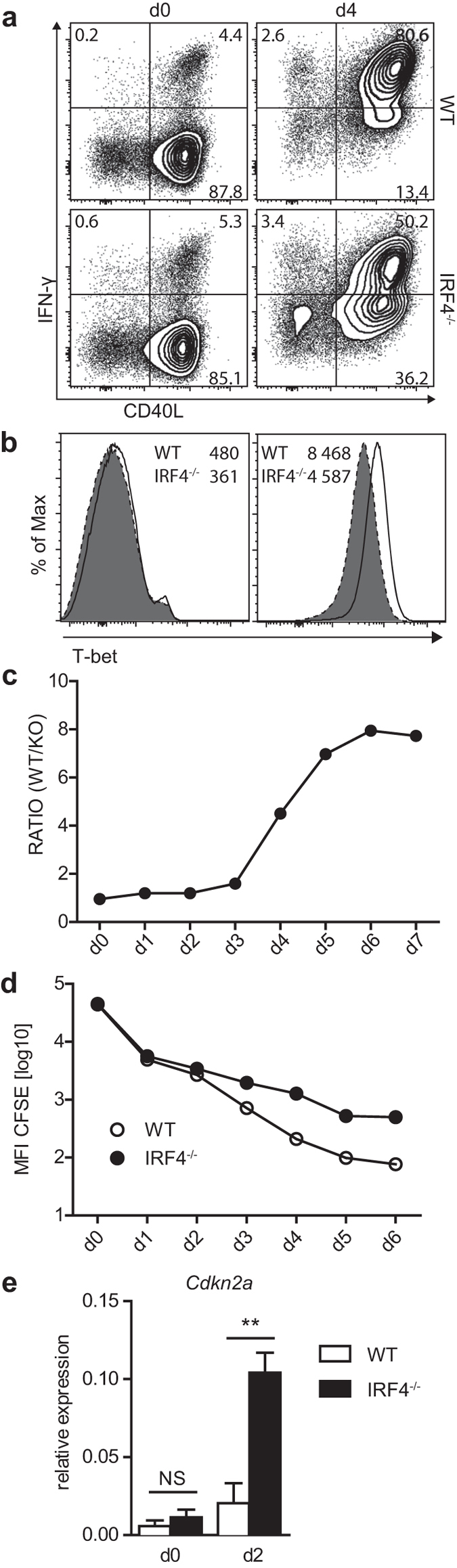
Impaired *in vitro* response of IRF4^−/−^ CD4^+^ T cells. CD4^+^ T cells from WT (CD90.1^+^) and IRF4^−/−^ mice (CD90.2^+^) were mixed with a 1:1 ratio. Cells were stimulated *in vitro* with anti-CD3 mAb, anti-CD28 mAb and IL-2 in the presence of IL-12 and anti-IL-4 mAb to induce T_H1_ differentiation. (**a**) After 4d, cells were stimulated for 4 h with PMA/ionomycin and IFN-γ and CD40L expression was determined by intracellular staining. Spleen cells from naive WT and IRF4^−/−^ mice were stimulated and stained in parallel (d0). (**b**) T-bet expression in WT and IRF4^−/−^ CD4^+^ T cells without stimulation and at d4 of stimulation (numbers give the MFIs). (**c**) Ratio of WT and IRF4^−/−^ CD4^+^ T cells in co-culture at indicated days of stimulation. (**d**) Loss of CFSE staining intensity on viable WT and IRF4^−/−^ CD4^+^ T cells. (**e**) Expression of *Cdkn2a* at d0 and d2 of purified WT and IRF4^−/−^ CD4^+^ T cells stimulated in individual cultures. Blots and histograms in (**a,b**) show representative results for CD4-gated cells. Results are representative for 3 (**a–c**) or 2 (**d**) independent experiments. Bars in (**e**) represent the mean ± SEM of 4 individual samples from 2 independent experiments.

To test whether absence of IRF4 prevents T_H1_ differentiation, WT and IRF4^−/−^ CD4^+^ T cells were mixed to obtain a ratio of 1:1, and polyclonally stimulated under T_H1_ cell inducing conditions. On day 4, cells were restimulated and tested for the expression of IFN-γ. Whereas 80% of WT CD4^+^ T cells expressed IFN-γ, only about 50% of IRF4^−/−^ CD4^+^ T cells were IFN-γ^+^ (Fig. 5a), and in these cells the mean IFN-γ expression level was lower than in the respective WT cells. In accordance with the impaired IFN-γ production, we could also observe diminished up-regulation of T-bet in IRF4^−/−^ CD4^+^ T cells (Fig. 5b). Thus, IRF4-deficiency impaired but did not generally prevent T_H1_ differentiation. Activated CD4^+^ T cells were also analyzed by quantitative PCR for the expression of *Runx3* and *Prdm1*, coding for two transcription factors involved in T-cell differentiation and representing targets of IRF4 in CD8^+^ T cells^25^,^26^. In contrast to CD8^+^ T cells, we observed similar induction for both *Runx3* and *Prdm1* in WT and IRF4^−/−^ CD4^+^ T cells (Figure S6a,b).

When co-cultures were analyzed for the ratio of WT to IRF4^−/−^ CD4^+^ T cells, we realized that over time WT CD4^+^ T cells vastly outnumbered IRF4^−/−^ CD4^+^ T cells (Fig. 5c). Compared to WT CD4^+^ T cells, IRF4^−/−^ CD4^+^ T cells also presented with diminished CD25 expression and reduced blast formation as indicated by a lower forward scatter (Figure S7a,b). To determine proliferation, WT and IRF4^−/−^ CD4^+^ T cells were mixed, labelled with CFSE, and the loss of CFSE staining was measured (Fig. 5d). Following activation, both WT and IRF4^−/−^ CD4^+^ T cells showed initial reduction of CFSE staining intensity. After 2–3 days, decrease of CFSE staining was halted in IRF4^−/−^ CD4^+^ T cells. In contrast, WT CD4^+^ T cells continued to lose staining intensity. A similar response was observed when WT and IRF4^−/−^ CD4^+^ T cells were activated in individual cultures (Figure S7c). In accordance with these observations, activated IRF4^−/−^ CD4^+^ T cells showed enhanced mRNA expression for the cyclin-dependent kinase inhibitor *Cdkn2a*, which has been identified as IRF4 target in CD8^+^ T cells^26^ (Fig. 5e).

*In vitro* activated CD4^+^ T cells were also analyzed for induction of apoptosis (Figure S8a). CD4^+^ T cells were stimulated and the proportion apoptotic was determined by measuring binding of the caspase substrate FLICA and uptake of propidium iodide (PI)^11^. Compared to WT controls, IRF4^−/−^ CD4^+^ T cells showed slightly reduced frequencies of early apoptotic (FLICA^+^PI^−^) cells and slightly enhanced frequencies of late apoptotic (FLICA^+^PI^+^) cells. Determination of mRNA expression of *Bcl2* and *Bcl2l11* coding for the anti-apoptotic protein BCL2 and the pro-apoptotic protein BIM, respectively, revealed similar induction of both mRNA species in activated WT and IRF4^−/−^ CD4^+^ T cells (Figure S8b,c). In summary, these results indicate that the impaired expansion of the IRF4^−/−^ CD4^+^ T cell population was mainly due to reduced proliferation and only to a minor degree to enhanced apoptosis.

### IRF4^−/−^ CD4^+^ T cells are impaired in cellular metabolism

**Figure 6:**
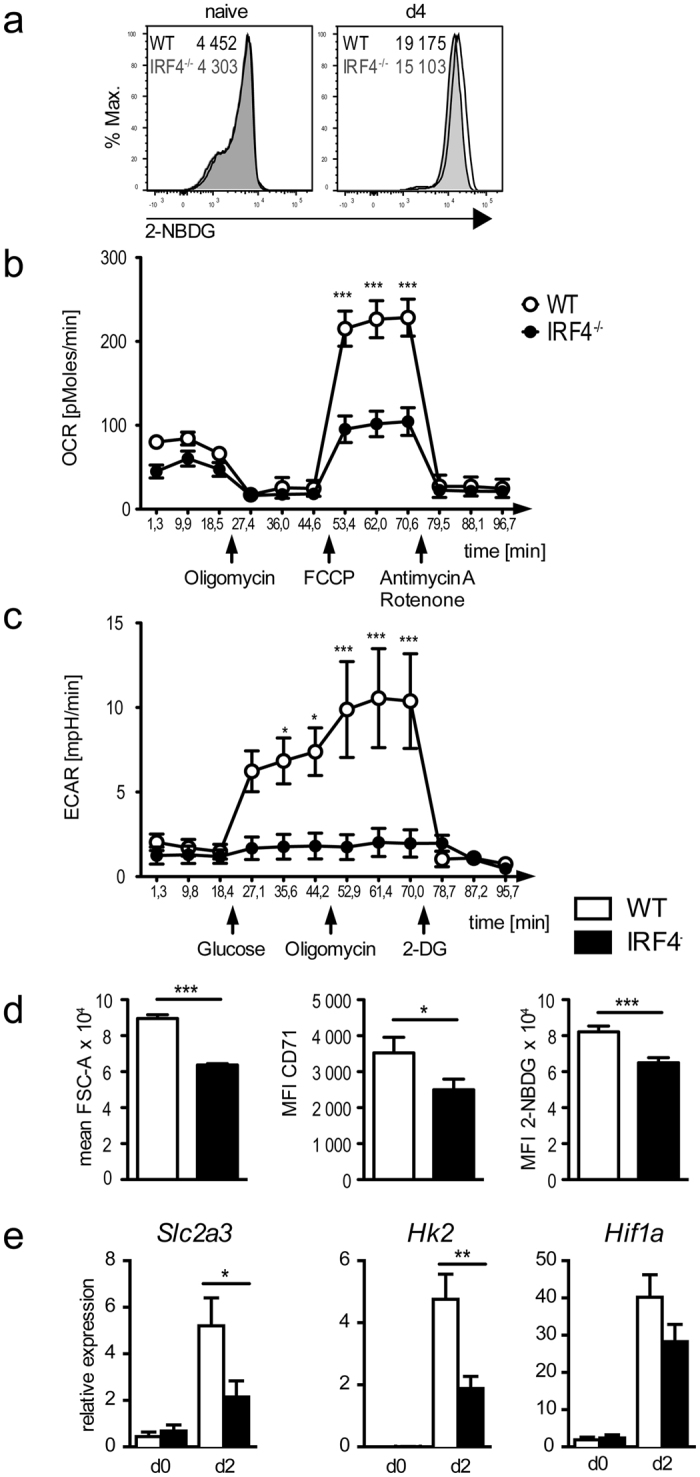
Altered metabolism in IRF4^−/−^ CD4^+^ T cells. (**a**) CD4^+^ T cells from WT (black lines) and IRF4^−/−^ mice (grey lines) were stimulated as described in Fig. 5. At day 4, cells were washed and in incubated with 30 μM 2-NBDG in glucose-free medium. After 2 h, 2-NBDG uptake was measured by flow cytometry. For comparison, spleen cells from naive mice (d0) were similarly analyzed (numbers give the MFIs). (**b,c**) CD4^+^ T cells were purified from spleens of WT and IRF4^−/−^ mice and stimulated over night with anti-CD3 mAb and anti-CD28 mAb. Cells were washed and OCR (**b**) and ECAR (**c**) at baseline level and in response to glucose and to different inhibitors were determined as described in the material method section. (**d**) Competitive transfer of WT and IRF4^−/−^ OT-II T cells was done as described in Fig. 4. Four days after transfer, OT-II cells from spleen were analyzed for size (FSC-A) and CD71 expression. Spleen cells were also in incubated with 30 μM 2-NBDG in glucose-free medium and after 2 h, 2-NBDG uptake was measured. (**e**) Expression of *Slc2a3, Hk2* and *Hif1a* in activated WT and IRF4^−/−^ CD4^+^ T cells. CD4^+^ T cells were purified and stimulated in individual cultures for 2d as described in Fig. 5. Histograms in (**a**) show CD4-gated cells and are representative for 3 independent experiments. Results in (**b,c**) are cumulative for 2 experiments with quintuplicate values for each cell subset. Bars in D represent mean ± SEM from 6 individually analyzed mice per group. Bars in (**e**) represent the mean ± SEM of 4 individual samples from 2 independent experiments.

For CD8^+^ T cells, it has been shown that IRF4 controls expression of proteins involved in central metabolic pathways such as glucose uptake, glycolysis and oxidative phosphorylation and thereby promotes changes in cell metabolism necessary for T cell proliferation and effector functions^25^. Recently it was also demonstrated that T_H1_ differentiation of CD4^+^ T cells is associated with similar metabolic changes^39^. To test whether IRF4 controls the metabolism of T_H1_ cells, WT and IRF4^−/−^ CD4^+^ T cells were activated and uptake of the fluorescent glucose analog 2-NBDG was measured by flow cytometry (Fig. 6a). Activation caused enhanced 2-NBDG uptake in WT and IRF4^−/−^ CD4^+^ T cells, but uptake was reduced in IRF4^−/−^ CD4^+^ T cells. The bioenergetics profile of IRF4^−/−^ T_H1_ cells was then tested with extracellular flux assays (Fig. 6b,c). Following activation, basal oxygen consumption rates (OCR) were slightly lower in IRF4^−/−^ than in WT CD4^+^ T cells (Fig. 6b). OCR was similar in both cell populations following treatment with the ATP synthase inhibitor oligomycin, however, IRF4^−/−^ CD4^+^ T cells showed impaired OCR increase in response to disruption of the proton gradient with FCCP, indicating that the maximal respiratory capacity was reduced in comparison to WT CD4^+^ T cells. The glycolytic capacity was tested by measuring extracellular acidification rates (ECAR). Compared to activated IRF4^−/−^ CD4^+^ T cells, activated WT CD4^+^ T cells showed an increase in ECAR after the addition of glucose. Blockade of ATP synthesis by oxidative phosphorylation with oligomycin further increased ECAR in WT CD4^+^ T cells, but had no effect on IRF4^−/−^ CD4^+^ T cells. Thus, deficiency of IRF4 in CD4^+^ T cells resulted in diminished glucose uptake and reduced glycolysis and oxidative phosphorylation.*Ex vivo* analysis of OT-II CD4^+^ T cells from competitive transfer assays supported the *in vitro* results. Four days after transfer, IRF4^−/−^ OT-II cells were smaller and expressed reduced levels of the transferrin receptor CD71 than WT OT-II cells (Fig. 6d). These IRF4^−/−^ OT-II cells also showed diminished ability to take up 2-NBDG. In line with these results and similar to CD8^+^ T cells^25^, we observed reduced mRNA expression for the glucose transporter GLUT3 (*Slc2a3*) and the central glycolysis enzyme hexokinase 2 (*Hk2*) in *in vitro* activated IRF4^−/−^ CD4^+^ T cells (Fig. 6e). Thus, IRF4 apparently controls metabolism in CD4^+^ T cells by regulating the expression of nutrient transporters and glycolysis enzymes. Interestingly and in contrast to IRF4^−/−^ CD8^+^ T cells^25^, we did not observe altered mRNA expression for the transcription factor HIF1α, a central regulator of cellular metabolism.

In conclusion, our results indicate that IRF4 is crucial for the regulation of metabolism in T_H1_ cells which results in a failure to mount T_H1_ responses.

## Discussion

Our study demonstrates that IRF4 is essential for effective formation of T_H1_ responses. Following *L. monocytogenes*-infection, listeria-specific IRF4^−/−^ CD4^+^ T cells showed impaired T_H1_ differentiation and failed to expand to the level observed for WT CD4^+^ T cells. Published analyses of the role of IRF4 in T_H1_ differentiation revealed inconsistent results^12^,^13^,^14^. *In vitro*, IRF4-deficient CD4^+^ T cells showed either normal T_H1_ differentiation^13^,^14^ or were significantly impaired^14^. Following *L. major* infection, studies described normal^13^ or severely diminished IFN-γ production^14^ when cells from lesion draining lymph nodes were stimulated with leishmania-antigen. In our study, we observed limited accumulation of specific IRF4^−/−^ CD4^+^ T cells in response to *L. monocytogenes* infection or peptide immunization. Remaining specific IRF4^−/−^ CD4^+^ T cells were impaired in T_H1_ differentiation as indicated by reduced production of IFN-γ and TNF-α (both in terms of frequency of responding cells and of cytokine expression per cell), diminished expression of T-bet and a failure to upregulate the T_H1_ markers CXCR3 and KLRG1. *In vitro*, IFN-γ production could be induced in IRF4^−/−^ CD4^+^ T cells, however, frequencies of IFN-γ^+^ cells were reduced and T-bet levels were diminished when compared to co-cultured WT cells. Thus, in contrast to the pronounced deficiency of IRF4^−/−^ CD4^+^ T cells to differentiate into T_H2_, T_H9_, T_H17_ and T_FH_ cells^12^,^13^,^14^,^15^,^16^,^17^,^18^,^19^,^20^, absence of IRF4 still allowed acquisition of some characteristics of T_H1_ cells. A more striking observation of our study was the pronounced failure of IRF4^−/−^ CD4^+^ T cells to accumulate to large populations *in vitro* and *in vivo*. In response to *L. monocytogenes* infection, we detected reduced accumulation of CD4^+^ T cells responding to listeria-antigen with the up-regulation of CD40L, and in competitive transfer experiments, IRF4^−/−^ CD4^+^ T cells were outnumbered by WT CD4^+^ T cells following infection or peptide immunization. Impaired accumulation of IRF4^−/−^ CD4^+^ T cells was also observed in co-culture experiments *in vitro*. This failure of expansion of IRF4^−/−^ CD4 T cells could also explain the inconsistent published results on IRF4 and T_H1_ differentiation^12^,^13^,^14^.

Impaired expansion of the IRF4^+^ CD4^+^ T cell population could be due to limited proliferation or a failure of survival and enhanced apoptosis. In contrast to a previous study^40^, we did not observe substantially enhanced apoptosis of IRF4^−/−^ CD4^+^ T cells when compared to WT cells in *in vitro* assays. In this study, apoptosis was determined after re-stimulation of highly activated CD4^+^ T cells, which might explain the different outcome when compared to our study, where apoptosis was measured following primary stimulation of naive CD4^+^ T cells. The small difference in apoptosis in our study was also consistent with the similar induction of the anti-apoptotic BCL2 and pro-apoptotic BIM in WT and IRF4^−/−^ CD4^+^ T cells. Our results rather indicate that IRF4^−/−^ CD4^+^ T cells have a defect in differentiation and proliferation. We observed diminished blast formation, reduced expression of activation markers and limited proliferation, as evident by less pronounced loss of CFSE staining. The *Cdkn2a* gene was identified as direct IRF4 target in CD8^+^ T cells and it was postulated that IRF4 acts as repressor of *Cdkn2a* expression and thereby allows proliferation of CD8^+^ T cells^26^. A similar mechanism could also be active in CD4^+^ T cells and restrict the proliferation of IRF4^−/−^ CD4^+^ T cells. In CD8^+^ T cells, IRF4 controls the adaption of the cellular metabolism to the requirements of proliferation and production of effector proteins following activation^25^,^33^. Activation of CD4^+^ T_H_ cells also causes profound changes of metabolic pathways including up-regulation of aerobic glycolysis^34^,^35^,^36^,^39^. Our results reveal that IRF4 is important for these changes in CD4^+^ T cells. Activated IRF4^−/−^ CD4^+^ T cells showed diminished surface expression of the transferrin receptor as well as reduced mRNA expression of the glucose transporter GLUT3 and impaired uptake of the glucose analog 2-NBDG. Cells had limited glycolytic capacity both at steady state and after blockade of mitochondrial ATP synthesis, which was associated with diminished hexokinase 2 expression. Finally, the maximal respiration capacity of activated IRF4^−/−^ CD4^+^ T cells was reduced. Thus, metabolic performance of activated IRF4^−/−^ CD4^+^ T cells was profoundly restricted and this limitation was likely responsible for the impaired response of IRF4^−/−^ CD4^+^ T cells.

Our data for IRF4^−/−^ T_H1_ cells resemble results for IRF4^−/−^ CD8^+^ T cells. These cells become activated but cannot sustain proliferation and effector function which eventually results in the collapse of the response. A similar scenario might occur during T_H1_ responses. IRF4^−/−^ CD4^+^ T cells become activated but fail to express genes required for proliferation and effector functions at adequate levels to allow effective T_H1_ differentiation and to sustain the response. We propose that this scenario is not restricted to T_H1_ differentiation but is also relevant for the deficiency of IRF4^−/−^ CD4^+^ T cells to differentiate into other T_H_ cell subsets. Thus, IRF4 is a fundamental transcription factor for all conventional T cells that controls adaptation of gene expression to the requirements of effector cells.

## Material and Methods

### Mice and infection

IRF4^−/−^ mice^8^, RAG1^−/−^ mice^41^, OT-II mice^42^ and CD90.1 congenic C57BL/6 mice (B6.PL-Thy1a/CyJ; Jackson, Bar Harbor, ME) were bred under specific pathogen-free conditions in the animal facility of the University Medical Center Hamburg-Eppendorf. Experiments were performed according to guidelines of the Deutsches Tierschutzgesetz and approved by the local committee for animal experiments of the City of Hamburg (permit number: 81/14). Mice were infected i.v. or i.p. with the indicated doses of either wild type *Listeria monocytogenes* (Lm) or of a *L. monocytogenes* strain recombinant for ovalbumin (LmOVA)^11^,^37^. Bacterial inocula were controlled by plating serial dilutions on tryptic soy broth (TSB) agar plates.

### Cell isolation and cell culture

Cells from spleens were obtained by mashing the disintegrated organs through cell sieves into PBS followed by erythrocyte lysis with ACK lysing buffer (155 mM NH_4_Cl, 10 mM KHCO_3_, 100 μM EDTA, pH ~7.2). For induction of cytokine production, cells were incubated at 1–5 × 10^6^ cells/ml in culture medium (RPMI 1640 medium supplemented with 5% fetal calf serum, glutamine, pyruvate, 2-mercaptoethanol and gentamicin) and stimulated for 4 h with 10^−5^ M ovalbumin peptide (OVA_323-339_; ISQAVHAAHAEINEAGR) or 10^−5^ M listeriolysin O peptide (LLO_189-201_; WNEKYAQAYPNVS) (both JPT Peptide Technologies, Berlin, Germany), or with 50 ng/ml phorbol 12-myristate 13-acetate (PMA, Sigma Aldrich, St. Louis, MO) and 1 μM ionomycin (Sigma Aldrich). 10 μg/ml brefeldin A (Sigma Aldrich) was added for the last 3.5 h of culture to prevent cytokine secretion. Subsequently cells were analyzed by flow cytometry^38^,^43^.

For *in vitro* T_H1_ cell differentiation, CD4^+^ T cells were purified using Mouse CD4^+^ T Cell Isolation Kit (Easy Sep, Stemcell Technologies, Vancouver, Canada). Cells were incubated for up to 7 days in 24-well plates coated with anti-CD3 (clone 145 2C11, Biolegend, San Diego, CA). Cultures were supplemented with 2 μg/ml anti-CD28 (37.51), 20 U/ml rhIL-2 (Novartis, Basel, Switzerland), and 10 ng/ml IL-12 (Biolegend). The medium was replaced depending on the pH-indicator. In some experiments, 2 μg/ml anti-IL-4 (11B11) was added to prevent cells from T_H2_ differentiation. In competitive cultures, CD4^+^ T cells from congenic CD90.1^+^ WT mice and CD90.2^+^ IRF4^−/−^ mice were mixed at a ratio of 1:1 and stimulated as described. For analysis of glucose uptake, CD4^+^ T cells from cell culture or directly isolated from infected mice were incubated for 2 h in glucose free RPMI 1640 medium (Life Technologies, Carlsbad, CA) with 30 μM 2-NBDG (2-[N-(7-nitrobenz-2-oxa-1,3-diazol-4-yl)amino]-2-deoxy-D-glucose; Life Technologies). Cells were washed, stained for surface markers and analyzed by flow cytometry. Proliferation was measured by loss of CFSE staining intensity and apoptosis was determined by FLICA and propidium iodide staining as described^11^.

mRNA expression was determined by RT-PCR as described previously^44^. Primer sequences are available upon request. All samples were run in triplicates. Results were normalized to the expression of 18S rRNA and are presented as relative expression, which was calculated as 2^−Δct^ × 1000.

### Flow cytometric analysis

For surface staining, cells were first incubated with 10 μg/ml anti-FcγRII/III (2.4G2; BioXCell, West Lebanon, NH) and 1:100 rat serum (Jackson Laboratories, Bar Harbor, ME) in PBS to minimize unspecific antibody binding. Staining was performed on ice with fluorochrome-conjugated mAb according to standard methods. Dead cells were labelled with a fixable dead cell stain (Pacific Orange succinimidyl ester; Life Technologies). Cells were measured on a CantoII flow cytometer (BD Biosciences, San Jose, CA) and data was analyzed with the FlowJo software (Treestar, Ashland, OR).

For detection of intracellular cytokines, cells were surface stained and then incubated with a Pacific Orange succinimidyl ester to exclude dead cells from analysis. Cells were washed with PBS and fixed for 15 min with PBS, 2% paraformaldehyde at room temperature. Thereafter, cells were washed with PBS, 0.2% BSA, permeabilized with PBS, 0.1% BSA, 0.3% saponin (Sigma, Aldrich), and incubated in this buffer with 1% rat serum. After 5 min, fluorochrome-conjugated antibodies were added. After further 15 min, cells were washed with PBS and measured by flow cytometry. T-bet staining was performed using the buffers of the Foxp3 Transcription Factor Staining Set (eBioscience, San Diego, CA).

Fluorochrome-labelled antibodies anti-CD4 (clone RM4-5), anti-CD8α (53-6.7), anti-CD25 (7D4), anti-CD44 (IM7), anti-CD62L (MEL-14), anti-CD71 (R17217.1.4), anti-CD90.1 (His51), anti-CD90.2 (53-2.1), anti-CD127 (A7R34), anti-CD154/CD40L (MR1), anti-KLRG1 (2F1), anti-TCR Vα2 (B20.1), anti-CXCR3 (CXCR3-173), anti-IFN-γ (XMG1.2), anti-TNF-α (MP6-XT22), anti-T-bet (eBio4B10) and anti-IRF4 (3E4) were purchased from BD Biosciences, BioLegend or eBioscience.

### Competitive T cell transfer assays

Spleen cells were isolated and CD4^+^ T cells were purified as described above. For competitive transfers in RAG1^−/−^ mice, purified CD4^+^ T cells from CD90.1^+^ congenic WT mice and CD4^+^ T cells from IRF4^−/−^ mice (CD90.2^+^) were mixed at a ratio of 1:1 and 2 × 10^6^ cells were i.v. injected into the RAG1^−/−^ mice, which had been infected i.p. 4 h before with 10^5^ Lm. Mice were analyzed at day 8 after T cell transfer. For competitive OT-II cell transfer, CD4^+^ T cells were purified from CD90.1^+^CD90.2^+^ OT-II WT mice (OT-II × B6.PL-Thy1a/CyJ F1 mice) and from CD90.2^+^CD90.1^−^ IRF4^−/−^ OT-II mice. Donor T cells were mixed at a ratio of 1:1 and 3 × 10^6^ cells were i.v. injected into CD90.1 congenic WT mice (CD90.1^+^CD90.2^−^). Recipient mice had either been i.v. infected with 10^5^ LmOVA one day before or were i.v. treated with 50 μg OVA_323-339_ peptide and 30 μg LPS 1 h post T cell transfer. Spleen cells from recipients were analyzed on day 4 after transfer.

### Extracellular flux assays

CD4^+^ T cells were purified from spleens of WT and IRF4^−/−^ mice and incubated for 18 h with the T cell expansion and activation kit based on anti-CD3 and anti-CD28 stimulation according to the manufacturer’s protocol (Miltenyi Biotec, Bergisch Gladbach, Germany). 4.5 × 10^5^ cells/well were transferred to analysis plates (Seahorse Bioscience, North Billerica, MA) coated with CellTak (Corning, Corning, NY, USA) and plates were centrifuged to accumulate cells at the bottom of the plates. For determination of oxygen consumption rates (OCR), cells were washed and incubated in XF assay medium, 1 mM sodium pyruvate and 25 mM glucose (Seahorse Bioscience). Cells were analyzed using a XF-24 Extracellular Flux Analyzer (Seahorse Bioscience) according to the manufacturer’s protocols. During OCR analysis, cells were treated with 1 μM oligomycin, 0.9 μM fluorocarbonyl cyanide phenylhydrazone (FCCP), 1 μM rotenone and 1 μM antimycin A (Seahorse Bioscience). For the analysis of extracellular acidification rates (ECAR), cells were pretreated as described above and incubated in analysis plates in glucose- and pyruvate-free DMEM 5030 (Sigma Aldrich). Cells were treated with 10 mM glucose, 1 μM oligomycin and 100 mM 2-DG (2-deoxyglucose, Seahorse Bioscience) during the assay. OCR and ECAR values were calculated using the program provided by the manufacturer.

### Statistical analysis

Statistical analyses were performed with Prism software (GraphPad Software Inc., La Jolla, CA, USA). Results were analyzed with the unpaired t test or in case of competitive cultures or transfers with a paired t test. A p-value of  <0.05 was considered significant (*p < 0.05; **p < 0.01; ***p < 0.001; ns: not significant).

## Additional Information

**How to cite this article**: Mahnke, J. *et al*. Interferon Regulatory Factor 4 controls T_H1_ cell effector function and metabolism. *Sci. Rep.* **6**, 35521; doi: 10.1038/srep35521 (2016).

## Supplementary Material

Supplementary Information
