# Supplementary material for: Interferon Regulatory Factor 4 controls TH1 cell effector function and metabolism
Source: Sci Rep. 2016 Oct 20;6:35521. doi: 10.1038/srep35521 (PMC5071867; doi:10.1038/srep35521)
Supplement: Supplementary Information [file srep35521-s1.pdf]

Supplementary File

Interferon Regulatory Factor 4 controls T_{H1} cell effector function and metabolism

Justus Mahnke[1], Valéa Schumacher[1], Stefanie Ahrens[1], Nadja Käding[2], Lea Marie Feldhoff[2], Magda Huber[3], Jan Rupp[2], Friederike Raczkowski[1#], Hans-Willi Mittrücker[1#*]

1) Institute of Immunology, University Medical Center Hamburg-Eppendorf, Hamburg, Germany

2) Department of Molecular and Clinical Infectious Diseases, University of Lübeck, Lübeck, Germany

3) Institute for Medical Microbiology and Hospital Hygiene, University of Marburg, Marburg, Germany

Supplementary Figures

Figure S1

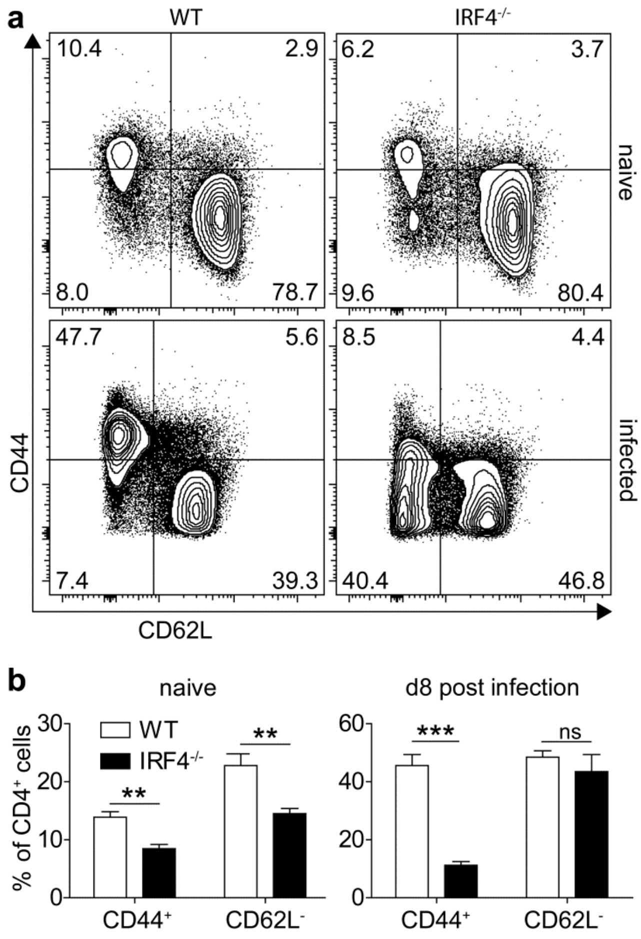

IRF4$^{-/-}$ mice show impaired CD4^{+} T cell response to infection with *L. monocytogenes*.

WT and IRF4$^{-/-}$ mice were i.v. infected with $5{\times}10^4$ LmOVA. Eight days post infection, CD4^{+} T cells from spleen were analyzed for CD44 and CD62L expression. (A) Representative dot plots for CD4-gated cells from naive and infected mice. (B) CD44 and CD62L expression of CD4^{+} T cells from naive and infected mice. Bars represent mean ± SEM from 4 individually analyzed mice per group. The result is representative for 2 independent experiments.

Figure S2

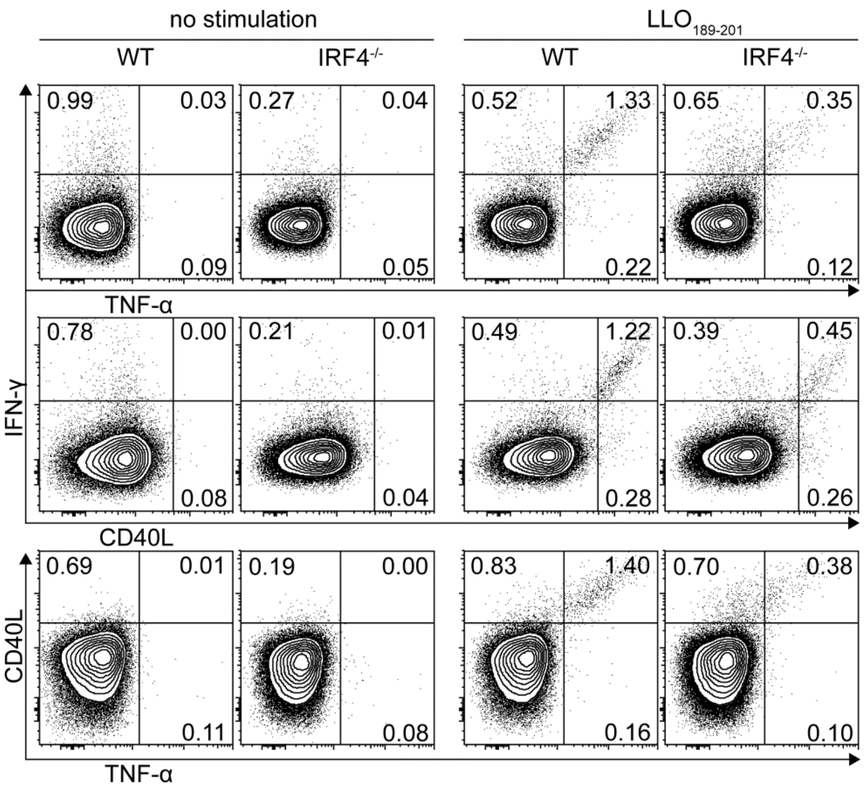

IRF4$^{-/-}$ mice fail to mount a T$_{H1}$ response against *L. monocytogenes*.

WT and IRF4$^{-/-}$ mice were i.v. infected with 5×10^4 *L. monocytogenes*. Eight days post infection, spleen cells were stimulated for 4h with 10^{-5}M LLO$_{189-201}$ or were incubated without stimulation. Intracellular expression of IFN-γ, TNF-α and CD40L was determined by flow cytometry. Dot blots show results for CD4-gated cells of infected WT and IRF4$^{-/-}$ mice. Results are representative for 4 individually analyzed mice per group and 2 independent experiments.

Figure S3

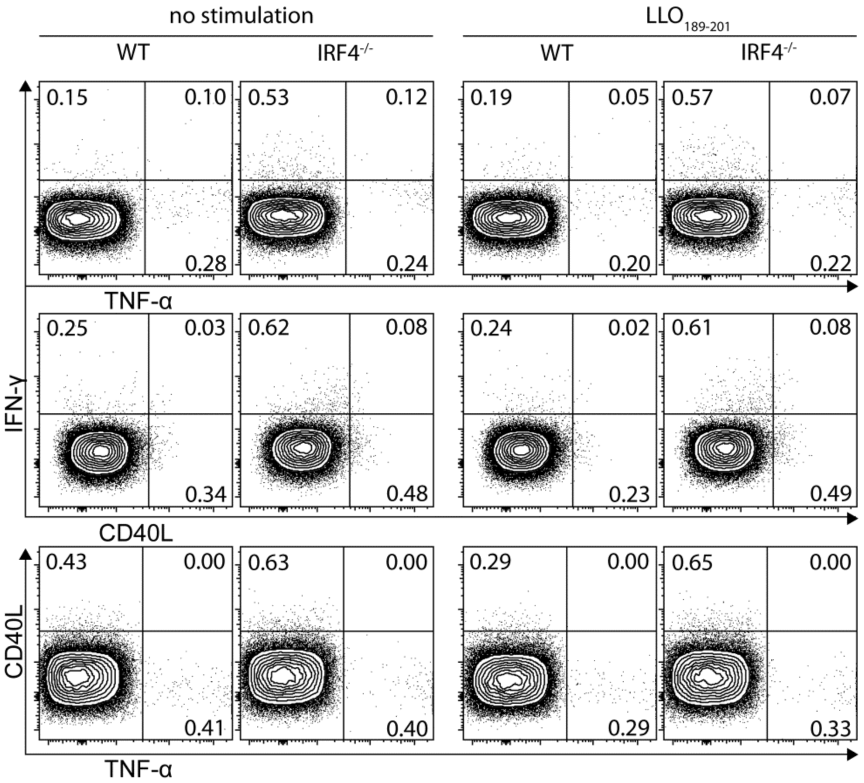

Control stimulation of spleen cells from naive WT and IRF4$^{-/-}$ mice.

Spleen cells from WT and IRF4$^{-/-}$ mice were stimulated for 4h with 10^{-5}M $LLO_{189-201}$ or were incubated without stimulation. Intracellular expression of IFN-γ, TNF-α and CD40L was determined by flow cytometry. Dot blots show results for CD4-gated cells of naive WT and IRF4$^{-/-}$ mice. Results are representative for 4 individually analyzed mice per group in one experiment.

Figure S4

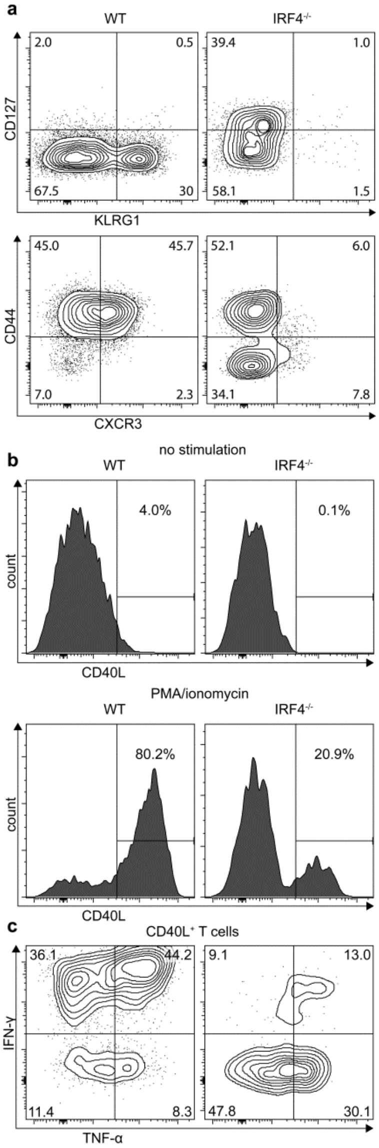

Impaired T_{H1} differentiation of IRF4$^{-/-}$ CD4^{+} T cells.

Purified CD4^{+} T cells from CD90.1^{+}CD90.2^{-} WT and CD90.1^{-}CD90.2^{+} IRF4$^{-/-}$ mice were mixed in a 1:1 ratio and 2×10^{6} cells were i.v. transferred into RAG1$^{-/-}$ mice, which had been infected with 10^{5} Lm 4h earlier. Eight days post infection, spleen cells of mice were analyzed. (A) Representative expression profile for CD127, KLRG1, CD44 and CXCR3 for CD90.1^{+}CD90.2^{-} WT CD4^{+} T cells and CD90.1^{-}CD90.2^{+} IRF4$^{-/-}$ CD4^{+} T cells. (B, C) Spleen cells from infected recipients were incubated for 4h with PMA/ionomycin or without stimulation and stained intracellularly for CD40L, TNF-α, and IFN-γ. (B) CD40L expression in CD4^{+} T cells. (C) IFN-γ and TNF-α expression in CD40L^{+} cells from stimulated WT and IRF4$^{-/-}$ CD4^{+} T cells. Representative results are shown.

Figure S5

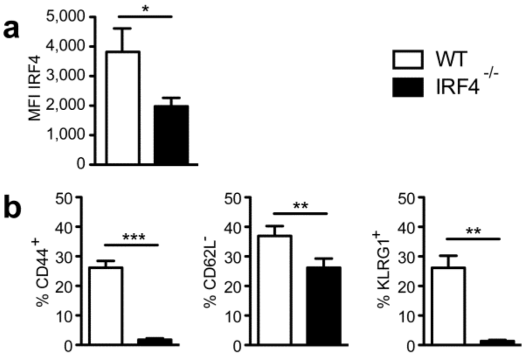

Impaired response of IRF4$^{-/-}$ OT-II cells.

Purified CD4^{+} T cells from WT CD90.1^{+}CD90.2^{+} or IRF4$^{-/-}$ CD90.1^{-}CD90.2^{+} OT-II mice were mixed at a 1:1 ratio and 3×10^{6} cells were i.v. transferred into CD90.1^{+}CD90.2^{-} WT mice. Recipient mice had been i.v. infected with 10^{5} LmOVA one day before. Spleen cells were analyzed 4d post transfer by extra- and intracellular mAb staining and flow cytometry. (A) IRF4 expression in WT and IRF4$^{-/-}$ OT-II cells. (B) Surface expression of CD44, CD62L and KLRG1 on WT and IRF4$^{-/-}$ OT-II cells. Bars give the mean ± SEM of 6 individually analyzed mice. Results are representative for 4 experiments.

Figure S6

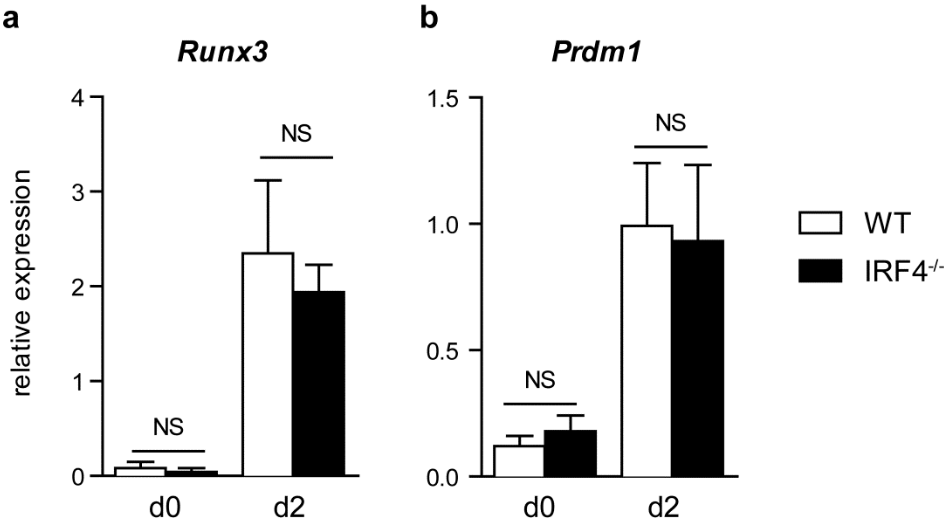

Expression of *Runx3* and *Prdm1* in activated WT and IRF4$^{-/-}$ CD4^{+} T cells.

CD4^{+} T cells were purified and stimulated in individual cultures for 2d as described in Figure 5. Expression of *Runx3* and *Prdm1* was measured by RT PCR. Bars represent the mean ± SEM of 4 individual samples from 2 independent experiments.

Figure S7

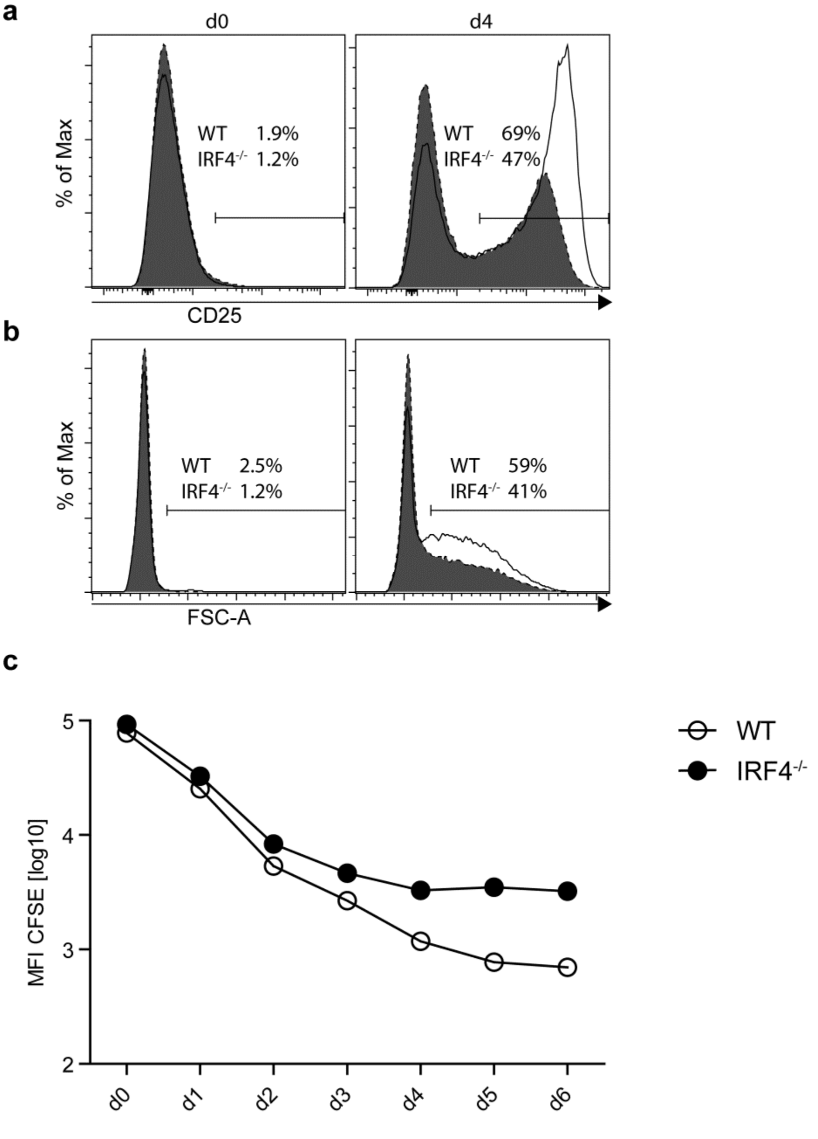

Phenotype and proliferation of activated WT and IRF4$^{-/-}$ CD4 T cells

CD4$^+$ T cells from WT (CD90.1$^+$) and IRF4$^{-/-}$ mice (CD90.2$^+$) were mixed with a 1:1 ratio and stimulated *in vitro* with anti-CD3 mAb, anti-CD28 mAb and IL-2 in the presence of IL-12 and anti-IL-4 mAb. (A) CD25 expression and (B) size (FSC-A) of WT and IRF4$^{-/-}$ CD4$^+$ T cells at d0 and d4 of stimulation. Histograms in (A) and (B) show representative results for CD4-gated cells. (C) CD4$^+$ T cells from WT and IRF4$^{-/-}$ mice were CFSE labelled and stimulated *in vitro* with anti-CD3 mAb, anti-CD28 mAb and IL-2 in the presence of IL-12 and anti-IL-4 mAb. CFSE staining intensity of viable cells was determined at the indicated days. Results are representative for 3 independent experiments (A, B) or one experiment (C).

Figure S8

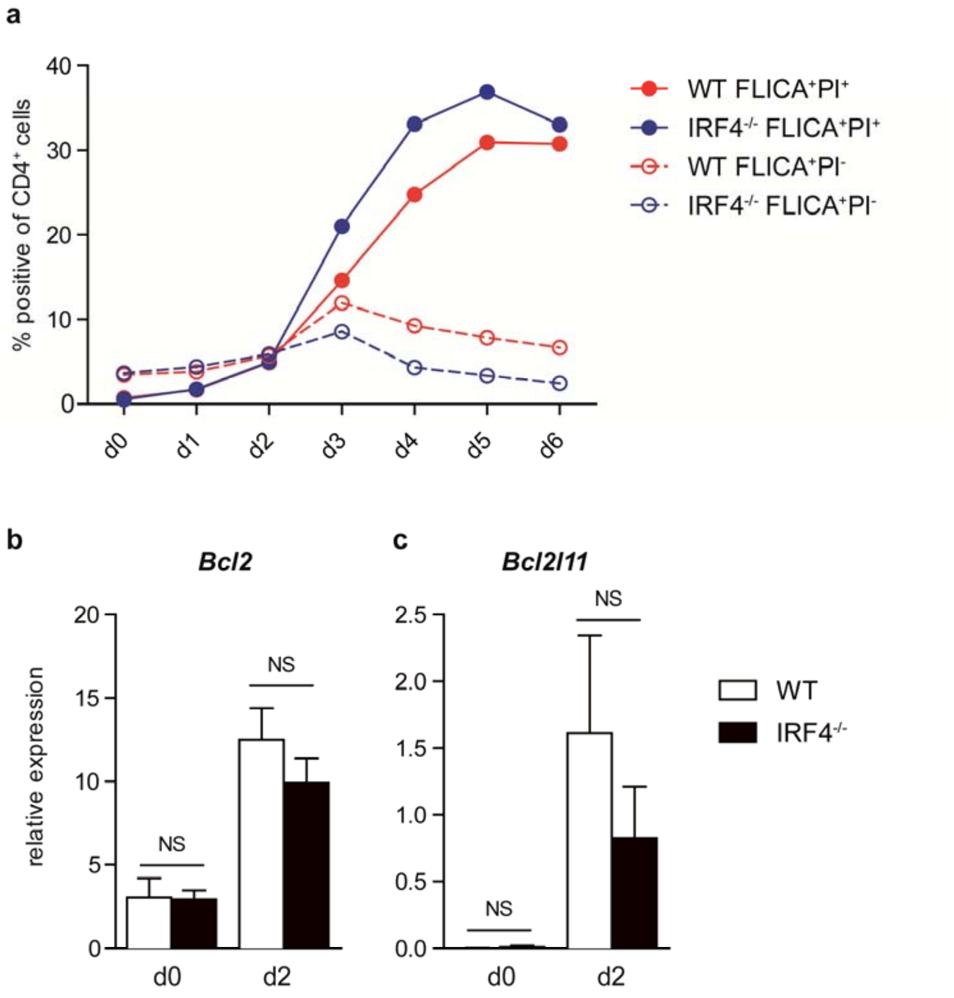

Apoptosis of WT and IRF4$^{-/-}$ CD4 T cells

CD4^{+} T cells from WT and IRF4$^{-/-}$ mice were stimulated in individual cultures with immobilized anti-CD3 mAb, soluble anti-CD28 mAb, IL-2 and IL-12. Frequencies of early (FLICA^{+}PI^{-}) and late apoptotic (FLICA^{+}PI^{+}) CD4^{+} T cells were determined at indicated days of culture. (B) Expression of *Bcl2* and *Bcl2l11* was measured by RT PCR. Result in (A) is representative for 2 independent experiments. Bars in (B) represent the mean ± SEM of 4 individual samples from 2 independent experiments.
